# Under-two child mortality according to maternal HIV status in Rwanda: assessing outcomes within the National PMTCT Program

**DOI:** 10.4314/pamj.v9i1.71215

**Published:** 2011-08-03

**Authors:** Placidie Mugwaneza, Nadine Wa Shema Umutoni, Hinda Ruton, Alphonse Rukundo, Alexandre Lyambabaje, Jean de Dieu Bizimana, Landry Tsague, Claire M Wagner, Elévanie Nyankesha, Jane Muita, Vincent Mutabazi, Jean Pierre Nyemazi, Sabin Nsanzimana, Corine Karema, Agnes Binagwaho

**Affiliations:** 1 Center for Treatment and Research on AIDS, Malaria, Tuberculosis and Other Epidemics, Kigali, Rwanda; 2 Department of Applied Mathematics, National University of Rwanda, Butare, Rwanda; 3 Development Research Group, World Bank, Washington DC, USA; 4 UNICEF Rwanda, Kigali, Rwanda; 5 Department of Global Health and Social Medicine, Harvard Medical School, Boston, USA; 6 Ministry of Health of Rwanda, Kigali, Rwanda

**Keywords:** HIV, PMTCT, maternal HIV infection, infant mortality, child mortality, under-five mortality, Rwanda

## Abstract

**Introduction:**

We sought to compare risk of death among children aged under-2 years born to HIV positive mother (HIV-exposed) and to HIV negative mother (HIV non-exposed), and identify determinants of under-2 mortality among the two groups in Rwanda.

**Methods:**

In a stratified, two-stage cluster sampling design, we selected mother-child pairs using national Antenatal Care (ANC) registers. Household interview with each mother was conducted to capture socio-demographic data and information related to pregnancy, delivery and post-partum. Data were censored at the date of child death. Using Cox proportional hazard model, we compared the hazard of death among HIV-exposed children and HIV non-exposed children.

**Results:**

Of 1,455 HIV-exposed children, 29 (2.0%; 95% CI: 1.3%-2.7%) died by 6 months compared to 18 children of the 1,565 HIV non-exposed children (1.2%; 95% CI: 0.6%-1.7%). By 9 months, cumulative risks of death were 3.0% (95%; CI: 2.2%-3.9%) and 1.3% (96%; CI: 0.7%-1.8%) among HIV-exposed and HIV non-exposed children, respectively. By 2 years, the hazard of death among HIV-exposed children was more than 3 times higher (aHR:3.5; 95% CI: 1.8-6.9) among HIV-exposed versus non-exposed children. Risk of death by 9-24 months of age was 50% lower among mothers who attended 4 or more antenatal care (ANC) visits (aHR: 0.5, 95% CI: 0.3-0.9), and 26% lower among families who had more assets (aHR: 0.7, 95% CI: 0.5-1.0).

**Conclusion:**

Infant mortality was independent of perinatal HIV exposure among children by 6 months of age. However, HIV-exposed children were 3.5 times more likely to die by 2 years. Fewer antenatal visits, lower household assets and maternal HIV seropositive status were associated with increased mortality by 9-24 months.

## Introduction

Mother-to-child transmission (MTCT) remains a major cause of pediatric human immunodeficiency virus (HIV) infection in sub-Saharan Africa, where more than 90% of the world's 2.1 million HIV-infected children live [1]. If not provided with timely and appropriate antiretroviral therapy (ART), about half of these children will die before they reach two years of age [2]. Children can be infected by HIV during gestation, birth, and through breastfeeding. Studies have reported the direct impact of HIV on maternal health and wellbeing, and its negative impact on child survival even in the absence of mother-to-child transmission of HIV [2, 3]. The use of antiretroviral drugs (ARV) in resource-limited settings has been proven effective both for prevention of mother-to-child transmission (MTCT) and treatment of adult and pediatric HIV diseases and is dramatically changing the face of the epidemic [4–12]. However, recent studies have established an increased risk of mortality among children who are exposed to HIV perinatally [13–18]. Until recently, few countries in sub-Saharan Africa had reported on the effectiveness of national PMTCT programs in terms of maternal and child survival benefits. Two studies in Malawi and Ghana reported on the effects of maternal HIV infection on child survival based on data from the 2003 Demographic and Health Survey [15, 19]. However, these did not reflect the era following the introduction of more efficacious ARV regimens in PMTCT guidelines in 2006, which had accelerated access to antiretroviral therapy [20, 21].

Globally, the under-five mortality rate has dropped from 89 deaths per 1,000 live births in 1990 to 60 in 2009. And yet, approximately 8.1 million children under the age of five died in 2009 alone, and 50% of those deaths occurred in sub-Saharan Africa [22]. With 129 deaths per 1,000 live births, sub-Saharan Africa's under-five mortality remains 20 times greater than the average in the developed world [22]. A multi-country pooled analysis indicated that HIV accounted for 5.4% and 3.6% of under-5 deaths in sub-Saharan Africa between 1990 and 2009, respectively, showing a decrease of the contribution of HIV to under-five mortality. This trend is very encouraging, but more efforts are required in order to achieve the fourth Millennium Development Goal (MDG4), which calls for a two-thirds reduction of the under-five mortality rate between 1990 and 2015, to which HIV is a contributing factor [22]. Rwanda has registered a dramatic decline in its under-five mortality rate from 152 to 103 deaths per 1000 live births within a five year period [23, 24].

Rwanda has an HIV prevalence estimated at 3% in the general population, accounting for an estimated 174,000 people, including 22,000 children [23, 25]. Were no interventions available to prevent mother-to-child transmission (PMTCT), 3,850 children among the 11,000 children born to the estimated 11,000 HIV-positive mothers in Rwanda would be infected with HIV [26]. The first PMTCT project in Rwanda began in 1999, followed by the National PMTCT program which was established in 2001 by the Ministry of Health. Since 2001, ARV regimens used in Rwanda have been in line with those recommended by the World Health Organization.

From 2001-2005, single dose Nevirapine (NVP) was utilized as the prophylaxis. In September 2005, Rwanda adopted the second WHO recommendations for PMTCT regimens based on dual therapy including Zidovudine (AZT) and Nevirapine, By 2009, the national PMTCT program's services could be accessed in 72% of all health facilities. An estimated 65% of HIV-positive pregnant women were receiving ART or ARV prophylaxis for PMTCT in 2009 [27], and about half were on dual ARV prophylaxis. The national PMTCT program implemented between 2005 and 2009 is described in further detail elsewhere [28]. In November 2010, Rwanda adopted the “Option B” of the 2010 WHO recommendations for the use of ARV in PMTCT; therefore, all HIV-positive pregnant women will receive triple ARV therapy (HAART) starting as soon as possible during pregnancy for all women eligible for lifelong triple therapy and from 14 weeks into pregnancy for women not eligible for lifelong treatment.

Although some underlying factors associated with under-five child mortality in Rwanda are known (e.g. mother's education, and mother's socio-economic status [23, 24]), little is known on the mortality outcomes of HIV-exposed children in Rwanda. The paucity of data on the contribution of HIV to current under-five mortality in Rwanda has stagnated the development and implementation of targeted interventions. In this study, we sought to analyze whether children born to HIV-positive mothers have a higher risk of mortality than children born to HIV-negative mothers; and to understand factors associated with mortality in order to inform national efforts to accelerate the reduction of child mortality. There is currently no indicator in Rwanda's Demographic and Health Survey on under-two mortality. The under-two mortality rate also contributes to the under-five mortality rate. As such, we employ under-five mortality as a proxy unit of comparison.

## Methods

### Study design

This retrospective cohort study is based on a cross-sectional survey of HIV-positive and HIV-negative mothers and their children.

### Study population

The participants included both mothers and children. Mother participants were HIV-positive or HIV-negative and expecting a child (or children) between March 2007 and June 2008; these only included mothers for whom at least one ANC visit was recorded. Maternal HIV status was determined by HIV rapid test and the national algorithm used at the time of the study, which included three tests: Determine, Unigold and Capillus. Child participants included the mothers’ children who were born during the aforementioned dates. Health facilities that were not providing PMTCT services, in addition to those who had not been providing PMTCT services for fewer than 36 months, were excluded from the study. Mothers who had not had at least one ANC visit and/or who were not expecting a child between the provided dates were excluded from participation. Children whose mothers were not participants in the study were excluded from the study.

A stratified two-stage cluster sampling method was used to select participants. First, health centers that had offered PMTCT services for at least 36 months prior to the survey were stratified dichotomously into urban and rural groups. Second, a random sample of health centers was selected from each stratum. Mothers were divided into three groups: HIV-positive mothers who had completed at least 4 ANC visits, HIV-positive mothers who did not complete 4 ANC visits; and HIV-negative mothers. As the recruitment was based on data from ANC registries, it was assumed that women with 4 ANC visits were more likely to comply with other PMTCT program activities than women who had not completed 4 ANC visits. Third, the mother-child pairs were randomly selected from each group and then tracked back to their homes for interviews. A transmission rate of 35% without intervention was assumed according to UNAIDS estimates [26], and a 10% reduction in transmission was assumed with intervention (25% transmission) given that estimates in Rwanda were not available at the time of the study. The 25% and 35% respective transmission rates allowed for a robust sample size calculation that met requirements for a 5% significance level, an 80% statistical power, an assumed 10% non-response rate, and a design effect of 2. Accordingly, a sample size of 3,420 mother-child pairs was required. The methodology was approved by the National Institute of Statistics of Rwanda.

### Data collection

At the household level, data were collected using two instruments: a questionnaire for mothers and a child health assessment card. The questionnaire was developed and the outcome and predictor variables were selected based on a literature review. The questionnaire was further refined in close collaboration with PMTCT professionals in Rwanda. Methods were pretested and revised accordingly. The questionnaire was administered in Kinyarwanda, the only local language in Rwanda. Interviews were conducted over the period of 45 minutes on average. This questionnaire was used to capture information about the knowledge of mothers about HIV (with a particular focus on PMTCT), the use of PMTCT services by HIV-positive women, and the use of reproductive health services. Several variables including number of ANC visits, ARVs taken by mother and child, place of delivery, and feeding options were measured using this questionnaire. The child health assessment card was used to record child basic demographics such as age and sex, overall health, as well as weight, height and HIV status.

The data presented in this article derive from a larger study on child health and HIV/AIDS in Rwanda. Blood samples were collected at the household level from child participants who were alive at the time of data collection. However, for the purposes of the present findings, and given that not all child participants were alive at the time of data collection, the child's HIV status was not included for the analysis in this article. Further, the cut-off of 9-24 months was chosen as part of the larger study on child health and HIV/AIDS in Rwanda wherein the child's HIV status between the ages 9-24 months was required for analysis. In Rwanda, the national PMTCT guidelines mandate the first HIV test for children born to HIV-infected mothers and HIV-discordant couples be administered at 6 weeks using the Polymerase Chain Reaction (PCR). The second and third tests are administered at 9 and 18 months, respectively, using the HIV rapid test. At the time of data collection, the test at 6 weeks had not been implemented in all health facilities that had been offering PMTCT services for the past 36 months. As such, the cut-off of 9 months was chosen to enable the HIV rapid test data to be used. The cut-off of 24 months was used according to PMTCT guidelines in Rwanda as a month wherein HIV-exposed children attend follow-up visits at the health facility.

### Data management and analysis

Completed questionnaires were periodically brought in from the field to the School of Public Health of the National University of Rwanda for data entry. Data were entered using CSPro 4.0. A quality control program was used to detect errors in data collection and data entry. This information was shared with field teams during supervisory visits and weekly meetings were held to improve data quality. In addition, 10% of the questionnaires were double-entered for data quality control at entry. Data was exported to STATA 10.1 for data analysis. We used descriptive statistics to describe essential demographic, social, and economic characteristics of participants.

### Primary study endpoint

The primary study endpoint was the cumulative risk of death among children 9-24 months old according to maternal HIV status. Secondary endpoints included social, and economic and behavioral factors that were associated with child mortality.

### Maternal predictor variables

The potential predictor variables included socio-demographic characteristics of the mother; indices for decision-making power about health and nutrition as well as decision-making power about short and long term investment strategies, housing material, household assets; in addition to household size, access to clean water, location of household, and ANC utilization.

**Figure 2:**
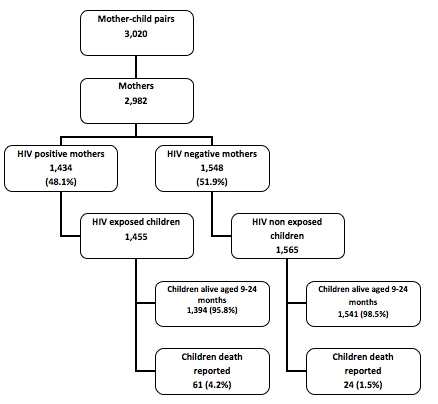
Kaplan –Meier survival curve of children born to HIV-negative and HIV-positive mothers

The risk of death among children born to HIV-infected mothers was compared to the risk of death among children of the same age born to HIV-negative mothers. As stated above, the purpose of this comparison was to determine if children born to HIV-infected mothers might be at higher risk of death than the rest of the children of the same age. All confidence intervals (CI) were at the 95% level of significance, and P-values of 0.05 and below were considered statistically significant. Kaplan-Meier survival analysis and log rank test were used to assess the difference in survival between children born to HIV-positive mothers and those born to HIV-negative mothers (Figure 2). We modeled the risk of dying by 9-24 months among children born to HIV-infected mothers using a Cox proportional hazard regression model. Categorical independent variables were coded as dummy variables in the regression model. Stepwise selection with a probability of 0.05 for a variable to enter the model and a probability of 0.15 to be removed from the model were used to test the model of the determinants of hazard of death among children. The final model included three variables: mother's HIV serostatus, the number of ANC visits, and ownership of assets index (Table 3).

### Ethical considerations

Appropriate measures were taken to ensure survey participant protection, signed and informed consent, voluntary participation and confidentiality. In addition, formal review and approval of the instruments were obtained from the Rwandan National Ethics Committee.

## Results

### Description of survey participants

**Figure 1:**
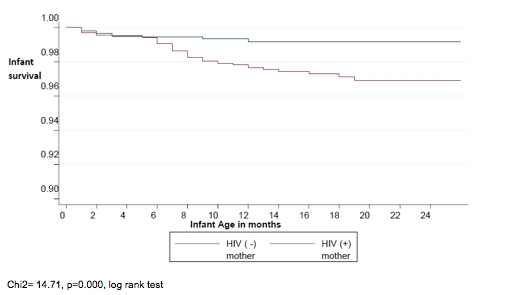
Profile of the survey participants (HIV-positive and negative mothers, HIV-exposed and non-exposed children)

One hundred and five health facilities were selected and visited. Of 3,020 children selected, 1,455 (48.2%) were HIV-exposed and 1,565 (51.8%) were not exposed to HIV (Figure 1). Twenty-six percent of HIV-positive mothers and 14% of HIV-negative mothers could not be interviewed for this study; the total non-response rate was 20%.

The mean ages of HIV-positive and HIV-negative mothers interviewed were 32.2 years and 30.1 years, respectively. The majority of HIV-positive and negative mothers (78.3% and 68.9%, respectively) were between 25 and 39 years old. Among all mothers, the majority were also living with a partner (68.9% of HIV-positive and 87.5% of HIV-negative mothers). Among HIV-positive mothers, 67% had a primary school education, and among HIV-negative mothers 69.4% had a primary school education. The study found that 55.1% of HIV-positive and 59.9% of HIV-negative mothers were able to read and write easily (Table 1). About 80% of mothers (HIV-positive or HIV-negative) lived in rural areas.

**Table 1:** Background Characteristics of HIV-positive and HIV-negative mothers expecting a child between March 2007 and June 2008, Rwanda, 2009

Characteristics		HIV-positive mothers		HIV-negative mothers	
		
		Number	%	Number	%
Mother's age (years)					
	15-24	143	10.0	330	21.5
	25-29	354	24.7	473	30.8
	30-34	415	29.0	347	22.6
	35-39	351	24.5	239	15.5
	40-44	141	9.9	120	7.8
	45-49	27	1.9	29	1.9
	Total	1,431		1,538	
Mean age		32.2		30.1
					
Marital Status	Single/Never married	122	8.5	97	6.3
	Lives with a partner	986	69.0	1,345	87.7
	Separated/Divorced/Widowed	321	22.5	92	6.0
	Total	1,429		1,534	
					
Religion	No religion	23	1.6	14	0.9
	Adventist	141	9.9	152	9.9
	Catholic	609	42.5	703	45.7
	Protestant	582	40.6	631	41.0
	Muslim	60	4.2	26	1.7
	Others	17	1.2	12	0.8
	Total	1,432		1,538	
					
Education					
	Never attended school	353	24.7	354	23.0
	Primary school	965	67.6	1,068	69.5
	Vocational/technical	37	2.6	27	1.8
	Secondary school	69	4.8	87	5.7
	University	4	0.3	1	0.1
	Total	1,428		1,537	
					
Literacy					
	cannot read and/or write; or has difficulty reading and/or writing	573	40.0	556	36.2
	cannot read and/or write; or has difficulty reading and/or writing	69	4.8	61	4.0
	can read and write easily	789	55.1	921	59.9
	Total	1,431		1,538	
					
Residence Location					
	Rural	1141	79.6	1,252	80.9
	Urban	293	20.4	296	19.1
Total		1,434		1,548	
					
Membership to a PLWH association	Yes	775	54.7		
	No	642	45.3		
	Total	1,417			
					
Housing index, (n=2882)		**Mean (SD)**			
	HIV+mothers	1.38(0.82)			
	HIV – mothers	1.43(0.82)			
				
Household asset index (n=2948)		**Mean (SD)**			
	HIV+mothers	0.89 (1.04)			
	HIV – mothers	1.16 (1.11)			

### Child mortality according to maternal HIV status

A total of 61 out of 1,455 children born to HIV-positive mothers died by 9-24 months compared to 24 out of 1,565 children born to HIV-negative mothers. The cumulative risk of death was almost three times higher in children born to HIV-positive mothers than in those who were born to HIV-negative mothers (4.2% vs. 1.5%; p

Twenty-nine out of 1,448 children born to HIV-positive mothers (2.0%; 95% Confidence Interval: 1.3%-2.7%) died before the age of 6 months compared to 18 out of 1,565 children born to HIV-negative mothers (1.2%; 95% CI: 0.6%-1.7%). No statistically significant difference in risk of dying by 6 months of age was observed between the two groups. By the age of 9 months, a total of 44 children out of 1,448 children born to HIV-positive mothers died; the cumulative risks of death were estimated at 3.0% (95% CI: 2.2%-3.9%) vs. 1.3% (95% CI: 0.7%-1.8%) for children born to HIV-positive and HIV-negative mothers, respectively. The difference in risk of dying by 9 months of age observed between the two groups was statistically significant (p=0.01).

Before the age of six months, mortality rates were similar in the two groups. However, after the age of six months, children born to HIV-positive mothers were at a higher risk of death than children born to HIV-positive mothers. According to the Kaplan-Meier survival analysis (Figure 2), children born to HIV-positive mothers were less likely to survive until 9-24 months than children born to HIV-negative mothers (p

### Maternal characteristics associated with death among under-2 years children

In univariate analysis, excluding maternal HIV status, household assets were significantly associated with death (Table 2). Ownership of assets was significantly lower on average in households that had lost the child (or children) compared to households whose child was (or children were) still alive at the time of the survey (mean assets: 0.8 vs. 1.0, p=0.033). The comparison of household assets to maternal or child HIV status was beyond the scope of this study. The remaining background characteristics (maternal age, marital status, literacy, and decision-making power) did not have a significant association with child mortality (Table 2).

**Table 2:** Cumulative mortality among HIV-exposed and non-exposed children by maternal and household characteristics

Maternal characteristics	Dead (%)	Alive (%)	P-value
HIV serological status (n=3,020)			
HIV-positive	4.2	95.8	<0.001
HIV-negative	1.5	98.5	
			
Age, years (n=3,007)			
15-24	3.2	96.8	
25-29	1.9	98.1	
30-34	3.0	97.0	
35-39	3.5	96.5	0.567
40-44	3.0	97.0	
45-49	3.6	96.4	
			
Marital status (n=3,001)			
Single/never married	4.0	96.0	
Lives with a partner	2.6	97.4	0.361
Separated/divorced/widowed	3.3	96.7	
			
Literacy (n=3,006)			
can't or have difficult reading and/or writing	3.2	96.9	
can read but can't or have difficult writing	3.8	96.2	0.492
can read and write easily	2.5	97.5	
			
Decision-making power^#^			
Health and nutrition issues means (n=3004)	90.65	89.48	0.590
Short and long term investment strategies means (n=3004)	83.43	82.95	0.872
			
Household characteristics Location,% (n=3020)			
Urban	3.4	96.6	
Rural	2.7	97.3	0.373
			
Housing index^@^, mean(SE) (n=2920)	1.3	1.4	0.357
Household assets index^$^, mean(SE) (n=2984)	0.8	1.0	0.033
			
Access to clean water,% (n=2966)			
Yes	2.7	97.3	0.596
No	3.1	96.9	

# Decision-making power about health and nutrition index is defined as a composite index created to measure the extent to which the respondent is involved in making household decisions related to health and nutrition issues. Scores ranged from 0 – 100 and a higher score indicates greater decision-making power. Decision-making power about short and longterm investment strategies index is defined as a composite index created to measure the extent to which the respondent is involved in making household decisions related to short and long-term investment issues. Scores ranged from 0 – 100 and a higher score indicates greater decision-making power.

@ Housing material index is defined as a composite index indicating whether roof, walls, and floors of the respondent's house is built in durable materials. The index ranged from 0 to 3; 0 meaning that none of the housing materials is durable while 3 meaning that materials for roof, walls and floors are durable.

$ Household asset index is defined as a composite index indicating the number of valuable equipments owned (Refrigerators, mobile telephone, radio, TV, car, etc).

**Table 3:** Cox proportional hazard regression models of time to death and factors associated with variability in time to death among under-2 years HIV-exposed and non-exposed children

	Adjusted Hazard ratio	95% CI
Mother's HIV status during pregnancy (reference: HIV-negative)		
HIV-positive	3.51	1.78– 6.89
		
Number of ANC visits (reference: fewer than 4)		
4 or more	0.50	0.26 – 0.93
		
Household asset index	0.74	0.54 – 1.03

In multivariate analysis (Table 3), the hazard of death among under-2 years children born to HIV-positive mothers was 3 times higher than it was among children born to HIV-negative mothers (adjusted Hazard Ratio; aHR: 3.5, 95% CI: 1.8-6.9). The hazard of death was 50% lower among children whose mothers had 4 or more ANC visits than among children whose mothers visited ANC services not more than once (aHR: 0.5, 95% CI: 0.3-0.9). Furthermore, the final model indicated that more assets owned by a household was associated with lower risk of death in children (aHR: 0.7, 95% CI: 0.5-1.0).

## Discussion

The present study is the first to report and compare mortality among HIV-exposed and HIV non-exposed children up to 2 years of age in a national PMTCT program in resource limited settings. The cumulative risk of death was 2.0% (95% CI: 1.3%-2.7%) by 6 months among HIV-exposed children and comparable to 1.2% (95% CI: 0.6%-1.7%) among HIV non-exposed children. By 9 months, the cumulative risk of death increased notably to 3.0% (95% CI: 2.2%-3.9%) among HIV-exposed children compared to 1.3% (96% CI: 0.7%-1.8%) among HIV non-exposed children. By 2 years, the cumulative risk of death reached 4.2% (95% CI: 2.2%-3.9%) among HIV-exposed children compared to 1.5% (96% CI: 0.7%-1.8%) among HIV non-exposed children. By 2 years, the hazard of death among HIV-exposed children was more than 3 times higher (aHR: 3.5, 95% CI: 1.8-6.9) compared to HIV non-exposed children. Risk of children death by 2 years of age was 50% lower among mothers who attended 4 or more antenatal care (ANC) visits (aHR: 0.5, 95% CI: 0.3-0.9), and 26% lower among families who had more household assets (aHR: 0.7, 95% CI: 0.5-1.0).

The mortality rate among HIV-exposed children in Rwanda is less than half of the rate reported from a pooled analysis of 3,468 HIV-exposed children in Africa showing an 11% mortality rate [2]. Although two years cumulative risk of death among HIV-exposed children is nearly three times higher than among HIV non-exposed children, it remains lower than the baseline infant mortality (6.2%) and under-five mortality rates (10.3%) in Rwanda [29].

In our study, maternal HIV status was associated with an increased risk of death (3.5 times) among under-two year children. Our findings are consistent with previous reports showing higher mortality (2.2-6.3 times) among children born to HIV-positive mothers compared to children born to HIV-negative mothers [2, 15, 30–33]. But no published studies reported mortality by 2 years, but mainly by 6 months. During the first 6 months of life, our study evidenced that mortality was low and independent from maternal HIV status. The low 6-month mortality (2.0%; 95% CI: 1.3%-2.7%) among HIV-exposed children from our study could be attributed in our context to the scaling up of various interventions including the PMTCT programs (roll-out of more efficacious ARV regimens including HAART for eligible pregnant women), promotion of exclusive breastfeeding for the first 4-6months, as well as increased access to cotrimoxazole [34] and ARV prophylaxis or treatment for HIV-exposed or infected children [35].

However, mortality significantly increased after 6 months among HIV-exposed children – a finding similar to those in other sub-Saharan African countries. These reports demonstrate early weaning at 4–6 months of age to be the most important and modifiable risk factor for infant mortality among HIV-exposed children after 6 months of age [36–39]. The infant feeding guidelines in Rwanda were aligned with the WHO guidelines of 2003 which recommended exclusive breastfeeding with rapid weaning at 4-6 months for HIV-exposed children when all the criteria to formula feed from birth were not met [21].

In a trial on the effect of early weaning on child health outcomes in Zambia, authors reported that children who died by 24 months were more likely to have mothers with low CD4 counts and high viral loads, and were more likely to have had mothers who died, as compared to children who did not die by 24 months [40]. This study demonstrated the important role maternal health plays as determinant of child survival. Without antiretroviral treatment, half of all HIV-infected children would die within the first 2 years of life [41]. Our study did not measure access to treatment for eligible women during pregnancy, nor did we assess early infection in children (6 weeks old) or children's HIV status at the time of death. However, we hypothesize that maternal health and treatment for HIV-infected children did not play a significant role on the increased child mortality after 6 months in our study. Shorter breast-feeding (or no breast-feeding) may increase mortality from common childhood illnesses [36, 39] and could be more detrimental for HIV-exposed but uninfected children. Mortality rates among HIV-exposed but uninfected children have been reported to exceed rates among HIV non-exposed children, even when feeding patterns are similar; intensive nutritional and counseling interventions reduce but do not eliminate this excess mortality [30, 31, 33, 40, 41].

From a public health standpoint, our findings support the recent revision of the PMTCT and infant feeding guidelines in Rwanda which promote improved maternal health, and extended breastfeeding with antiretroviral prophylaxis during 18 months [42]. Exclusive breastfeeding of HIV-exposed children is recommended for the first six months with the introduction of healthy, balanced, and appropriate complementary food at six months and continuation of breastfeeding without exceeding the maximum recommended duration of 18 months. Weaning should be done gradually over a period of one month in conjunction with advice and nutritional support.

In our study, higher frequency of ANC visits is associated with lower risk of death in children. Similarly, in a study conducted in Ghana, children born to mothers who did not receive antenatal care were 1.7 times more likely to die before reaching their first birthday than children of mothers who did receive antenatal care [15]. Receiving prenatal care was also found to be associated with lower risk of infant mortality in Bangladesh [43].

Another significant factor in child mortality evidenced in our study and in the current literature concerns household assets. The present research found that more assets owned by a household are associated with lower risk of death in children. Similarly, a study conducted in Cambodia revealed that children born in the poorest 40% of households were more than twice as likely to die during infancy as those born in the richest 20% of households [44]. During a clinical trial on child health outcomes in Zambia, authors reported that children who died were more likely to have lived in households reporting food insecurity and with more than one child under 5 years of age [40]. A number of studies found an association with maternal health care seeking behavior and household wealth. In a study conducted in Bangladesh among rural women, 22% of those in the lowest wealth quintile and 69% in the highest quintile reported having sought antenatal care from a medically trained provider [45]. Another study in Bangladesh found that low household asset status was a major determinant of health-seeking behavior [46].

Our analysis was based on data collected from a household survey of mothers selected from ANC registers in PMTCT sites. Even those women who had had only one ANC visit were included in the study sample. According to the 2007-08 Rwanda Interim Demographic and Health Survey, 94% of women who had live births in the past five years received ANC [24] from trained health personnel at least once. Thus, our sample was drawn from 94% of pregnant women in Rwanda. Given that 73% of women attending ANC visit were offered HIV testing in Rwanda [47], we were able to identify our sample from 68% of the all mothers who delivered a live birth infant from March 2007 to June 2008.

Our study is representative of all women who had attended at least one ANC visit in PMTCT program sites, and we used a robust two-stage cluster sampling approach. Yet we excluded some health facilities that had been offered PMTCT services for less than 36 months. Mothers who did not consult ANC services were also excluded from the study, and thus did not represent all expecting women during this timeframe. As part of the larger study from which these data on mortality derived, HIV tests were administered at the household level to those children who were still alive at the time of the study. However, given the retrospective nature of the study, many child participants had died at the time of the data collection. Therefore, it was not possible to link the child's death to his or her HIV status. It is uncertain as to whether mothers completely stopped breastfeeding at 9 months, which could confound the data analysis on weaning and survival. Although most mothers responded to all questions, the absence of responses from some participating mothers is an additional limitation. Finally, recall bias concerning PMTCT service use may have influenced study findings.

Further studies should be conducted to fill current gaps in the literature. A longitudinal study on a cohort of children born to HIV-positive and HIV-negative mothers who come for infant immunization at 6 weeks, and HIV tests and follow-up visits until 24 months, would be beneficial. This would diminish recall bias and fit into the regular health visits of a family. In addition, a multivariate analysis comparing ANC utilization to maternal characteristics would be a valuable addition to current scientific literature on child mortality.

## Conclusion

Exposure to HIV has been recently reported to contribute to 3.6-4.0% of under-five mortality in sub-Saharan Africa [48, 49]. In Rwanda, this would translate into 1,200 under-five deaths due to HIV out of the 41,200 under-five deaths from a hypothetical 400,000 annual live births. The prognosis for HIV-exposed children is comparable to that of HIV non-exposed children before 6 months of age. However, the risk of dying is significantly higher by 9-24 months old among HIV-exposed children. All HIV-exposed children should be targeted with child survival interventions in order to decrease the mortality differential existing between HIV-exposed and HIV non-exposed children. To reduce infant and child mortality, particularly HIV-related mortality, it is necessary to ensure that the revised infant feeding guidelines and communication strategies be implemented to prevent continuation of abrupt weaning practices. Rwanda will also need to increase the demand for and access to ANC visits by mothers. Further, integration of targeted social protection strategies will promote equity in access to effective child survival interventions for children born into families with a low household asset score. Our findings provide data required for Rwanda to better guide programmatic interventions targeting child mortality in the context of HIV/AIDS.

## References

[1] WHO/UNAIDS/UNICEF (2008). Towards universal access: scaling up priority HIV/AIDS interventions in the health sector.

[2] Newell ML, Brahmbhatt H, Ghys PD (2004). Child mortality and HIV infection in Africa: a review. AIDS.

[3] Newell ML, Coovadia H, Cortina-Borja M, Rollins N, Gaillard P, Dabis F (2004). Mortality of infected and uninfected infants born to HIV-infected mothers in Africa: a pooled analysis. Lancet.

[4] de Martino M, Tovo PA, Balducci M, Galli L, Gabiano C, Rezza G, Pezzotti P (2000). Reduction in mortality with availability of antiretroviral therapy for children with perinatal HIV-1 infection- Italian Register for HIV Infection in Children and the Italian National AIDS Registry. JAMA.

[5] Antiretroviral Drugs for Treating Pregnant Women and Preventig HIV Infection in Infants Towards Universal Access. Recommendations for a Public Health Approach. http://www.who.int/hiv/pub/guidelines/pmtctguidelines2.

[6] WHO/UNAIDS/UNICEF (2009). Towards universal access: scaling up priority HIV/AIDS interventions in the health sector.

[7] Gibb DM, Duong T, Tookey PA, Sharland M, Tudor-Williams G, Novelli V, Butler K, Riordan A, Farrelly L, Masters J (2003). Decline in mortality, AIDS, and hospital admissions in perinatally HIV-1 infected children in the United Kingdom and Ireland. BMJ.

[8] Puthanakit T, Aurpibul L, Oberdorfer P, Akarathum N, Kanjananit S, Wannarit P, Sirisanthana T, Sirisanthana V (2007). Hospitalization and mortality among HIV-infected children after receiving highly active antiretroviral therapy. Clin Infect Dis.

[9] George E, Noel F, Bois G, Cassagnol R, Estavien L, Rouzier Pde M, Verdier RI, Johnson WD, Pape JW, Fitzgerald DW (2007). Antiretroviral therapy for HIV-1-infected children in Haiti. J Infect Dis.

[10] Fassinou P, Elenga N, Rouet F, Laguide R, Kouakoussui KA, Timite M, Blanche S, Msellati P (2004). Highly active antiretroviral therapies among HIV-1-infected children in Abidjan, Cote d'Ivoire. AIDS.

[11] Farmer P, Leandre F, Mukherjee JS, Claude M, Nevil P, Smith-Fawzi MC, Koenig SP, Castro A, Becerra MC, Sachs J (2001). Community-based approaches to HIV treatment in resource-poor settings. Lancet.

[12] De Baets AJ, Bulterys M, Abrams EJ, Kankassa C (2007). Pazvakavambwa IE. Care and treatment of HIV-infected children in Africa: issues and challenges at the district hospital level. Pediatr Infect Dis J.

[13] Zaba B, Whitworth J, Marston M, Nakiyingi J, Ruberantwari A, Urassa M, Issingo R, Mwaluko G, Floyd S, Nyondo A (2005). HIV and mortality of mothers and children: evidence from cohort studies in Uganda, Tanzania, and Malawi. Epidemiology.

[14] Verhoeff FH, Le Cessie S, Kalanda BF, Kazembe PN, Broadhead RL, Brabin BJ (2004). Post-neonatal infant mortality in Malawi: the importance of maternal health. Ann Trop Paediatr.

[15] Hong R, Banta JE, Kamau JK (2007). Effect of maternal HIV infection on child survival in Ghana. J Community Health.

[16] Nakiyingi JS, Bracher M, Whitworth JA, Ruberantwari A, Busingye J, Mbulaiteye SM, Zaba B (2003). Child survival in relation to mother's HIV infection and survival: evidence from a Ugandan cohort study. AIDS.

[17] Crampin AC, Floyd S, Glynn JR, Madise N, Nyondo A, Khondowe MM, Njoka CL, Kanyongoloka H, Ngwira B, Zaba B (2003). . The long-term impact of HIV and orphanhood on the mortality and physical well-being of children in rural Malawi. AIDS.

[18] Ng'weshemi J, Urassa M, Isingo R, Mwaluko G, Ngalula J, Boerma T, Marston M, Zaba B (2003). HIV impact on mother and child mortality in rural Tanzania. J Acquir Immune Defic Syndr.

[19] Hong R (2008). Association of maternal HIV infection with increase of infant mortality in Malawi. J Paediatr Child Health.

[20] Antiretroviral Drugs for Treating Pregnant Women and Preventig HIV Infection in Infants (2006). Towards Universal Access. Recommendations for a Public Health Approach. http://www.who.int/hiv/pub/guidelines/pmtctguidelines2.

[21] World Health Organisation (WHO) (2003). HIV and infant feeding – Guidelines for decision-makers.

[22] United Nations Inter agency Group for Child Mortality Estimation (2010). Levels & Trends in Child Mortality.

[23] Institut National de la Statistique du Rwanda (INSR) and ORC Macro (2005). Rwanda Demographic and Health Survey.

[24] Ministry of Health (MOH) [Rwanda] Rwanda Interim Demographic and Health Survey 2007-08.

[25] Center for Treatment and Research on AIDS Malaria Tuberculosis and Other Epidemics Rwanda (2010). HIV and AIDS in Rwanda.

[26] Joint United Nations Programme on HIV/AIDS (1999). Prevention of HIV transmission from mother to child: Strategic options.

[27] Joint United Nations Programs on HIV/AIDS (UNAIDS) & WHO (2010). UNAIDS Report on the Global AIDS Epidemic 2010.

[28] Tsague L, Tsiouris FO, Carter RJ, Mugisha V, Tene G, Nyankesha E, Koblavi-Deme S, Mugwaneza P, Kayirangwa E, Sahabo R (2010). Comparing two service delivery models for the prevention of mother-to-child transmission (PMTCT) of HIV during transition from single-dose nevirapine to multi-drug antiretroviral regimens. BMC Public Health.

[29] Ministry of Health (2008).

[30] Marinda E, Humphrey JH, Iliff PJ, Mutasa K, Nathoo KJ, Piwoz EG, Moulton LH, Salama P, Ward BJ (2007). Child mortality according to maternal and infant HIV status in Zimbabwe.. Pediatr Infect Dis J.

[31] Brahmbhatt H, Kigozi G, Wabwire-Mangen F, Serwadda D, Lutalo T, Nalugoda F, Sewankambo N, Kiduggavu M, Wawer M, Gray R (2006). Mortality in HIV-infected and uninfected children of HIV-infected and uninfected mothers in rural Uganda. J Acquir Immune Defic Syndr.

[32] Lallemant C, Halembokaka G, Baty G, Ngo-Giang-Huong N, Barin F, Le Coeur S (2010). Impact of HIV/Aids on Child Mortality before the Highly Active Antiretroviral Therapy Era: A Study in Pointe-Noire, Republic of Congo. J Trop Med..

[33] Shapiro RL, Lockman S, Kim S, Smeaton L, Rahkola JT, Thior I, Wester C, Moffat C, Arimi P, Ndase P (2007). Infant morbidity, mortality, and breast milk immunologic profiles among breast-feeding HIV-infected and HIV-uninfected women in Botswana. J Infect Dis.

[34] Mulenga V, Ford D, Walker AS, Mwenya D, Mwansa J, Sinyinza F, Lishimpi K, Nunn A, Gillespie S, Zumla A (2007). Effect of cotrimoxazole on causes of death, hospital admissions and antibiotic use in HIV-infected children. AIDS.

[35] UNICEF (2010). Children and AIDS Fifth Stocktaking Report. UNICEF.

[36] Kuhn L, Aldrovandi GM, Sinkala M, Kankasa C, Semrau K, Mwiya M, Kasonde P, Scott N, Vwalika C, Walter J (2008). Effects of early, abrupt weaning on HIV-free survival of children in Zambia. N Engl J Med.

[37] Kafulafula G, Hoover DR, Taha TE, Thigpen M, Li Q, Fowler MG, Kumwenda NI, Nkanaunena K, Mipando L, Mofenson LM (2010). Frequency of gastroenteritis and gastroenteritis-associated mortality with early weaning in HIV-1-uninfected children born to HIV-infected women in Malawi.. J Acquir Immune Defic Syndr.

[38] Homsy J, Moore D, Barasa A, Were W, Likicho C, Waiswa B, Downing R, Malamba S, Tappero J, Mermin J (2010). Breastfeeding, mother-to-child HIV transmission, and mortality among infants born to HIV-Infected women on highly active antiretroviral therapy in rural Uganda. J Acquir Immune Defic Syndr.

[39] Thior I, Lockman S, Smeaton LM, Shapiro RL, Wester C, Heymann SJ, Gilbert PB, Stevens L, Peter T, Kim S (2006). Breastfeeding plus infant zidovudine prophylaxis for 6 months vs formula feeding plus infant zidovudine for 1 month to reduce mother-to-child HIV transmission in Botswana: a randomized trial: the Mashi Study. Jama.

[40] Kuhn L, Sinkala M, Semrau K, Kankasa C, Kasonde P, Mwiya M, Hu CC, Tsai WY, Thea DM (2010). Aldrovandi GM. Elevations in mortality associated with weaning persist into the second year of life among uninfected children born to HIV-infected mothers. Clin Infect Dis.

[41] Newell ML, Coovadia H, Cortina-Borja M, Rollins N, Gaillard P, Dabis F (2004). Ghent International ASWGoHIVIiWaC. Mortality of infected and uninfected infants born to HIV-infected mothers in Africa: a pooled analysis-[see comment]. Lancet.

[42] World Health Organisation (WHO) (2010). Antiretroviral drugs for treating pregnant women and preventing HIV infection in infants-Recommendations for a public health approach 2010 version.

[43] Hong R (2006). Effect of multiple birth on infant mortality in Bangladesh. J Paediatr Child Health.

[44] Hong R, Mishra V, Michael J (2007). Economic disparity and child survival in Cambodia. Asia Pac J Public Health.

[45] Koenig MA, Jamil K, Streatfield PK, Saha T, Al-Sabir A, El Arifeen S, Hill K, Haque Y (2007). Maternal health and care-seeking behavior in Bangladesh: findings from a national survey. Int Fam Plan Perspect.

[46] Amin R, Shah NM, Becker S (2010). Socioeconomic factors differentiating maternal and child health-seeking behavior in rural Bangladesh: A cross-sectional analysis. Int J Equity Health.

[47] Asiimwe A, Delvaux T, Elul B, Ndagije F, Roberfroid D, Munyana E, Nyiransabimana M, Mugenzi C, Vant Pad J, Mugisha V (2007).

[48] Stanecki K, Daher J, Stover J, Akwara P, Mahy M (2010). Under-5 mortality due to HIV: regional levels and 1990-2009 trends. Sex Transm Infect.

[49] Black RE, Cousens S, Johnson HL, Lawn JE, Rudan I, Bassani DG, Jha P, Campbell H, Walker CF, Cibulskis R (2010). Global, regional, and national causes of child mortality in 2008: a systematic analysis. Lancet.

